# A proof-of-concept study with the tyrosine kinase inhibitor nilotinib in spondyloarthritis

**DOI:** 10.1186/s12967-016-1050-2

**Published:** 2016-10-27

**Authors:** Jacqueline E. Paramarta, Maureen C. Turina, Troy Noordenbos, Tanja F. Heijda, Iris C. Blijdorp, Nataliya Yeremenko, Dominique Baeten

**Affiliations:** 1 Department of Clinical Immunology and Rheumatology, Academic Medical Center/University of Amsterdam, Meibergdreef 9, 1105 AZ Amsterdam, The Netherlands; 2 Laboratory of Experimental Immunology, Academic Medical Center/University of Amsterdam, Meibergdreef 9, 1105 AZ Amsterdam, The Netherlands

**Keywords:** Spondyloarthritis, Nilotinib, Tyrosine kinase inhibitor, Randomized controlled trial, Mast cells

## Abstract

**Background:**

To evaluate the immunomodulating and clinical effects of nilotinib, a tyrosine kinase inhibitor, in a proof-of-concept study in spondyloarthritis (SpA) assessing the mast cell as potential novel therapeutic target in this disease.

**Methods:**

Twenty eight patients with active peripheral (pSpA) and/or axial SpA (axSpA) were included in a randomized, double-blind, placebo-controlled clinical trial (Trial registration: Trialregister.nl NTR2834). Patients were treated 1:1 with nilotinib or placebo for 12 weeks, followed by an open label extension for another 12 weeks. Paired synovial tissue biopsies, serum sampling and assessment of clinical symptoms were performed serially.

**Results:**

In pSpA (n = 13) synovial inflammation appeared to diminish after 12 weeks of nilotinib treatment as evidenced by histopathology (decrease in number of infiltrating CD68+ and CD163+ macrophages and mast cells). Compared to placebo mRNA expression of c-Kit as mast cell marker (p = 0.037) and of pro-inflammatory cytokines such as IL-6 (p = 0.024) were reduced. The reduction of synovial inflammation was paralleled by a decrease in serum biomarkers of inflammation such as C-reactive protein (p = 0.024) and calprotectin (p = 0.055). Also clinical parameters such as patient’s global assessment of disease activity (p = 0.031) and ankylosing spondylitis disease activity score (p = 0.031) showed improvement upon 12 weeks of nilotinib but not placebo treatment. This improvement was further augmented at week 24. In contrast to pSpA, neither serum biomarkers of inflammation nor clinical parameters improved upon nilotinib treatment in axSpA. During the trial one serious adverse event occurred, which was considered unrelated to the study drug.

**Conclusions:**

This small proof-of-concept study suggests that nilotinib treatment modulates inflammation and clinical symptoms in pSpA. A similar effect was not seen in axSpA.

*Trial registration:* trialregister.nl registration code NTR2834 registered 31 March 2011

## Background

The introduction of tumor necrosis factor (TNF) inhibitors has improved the management of spondyloarthritis (SpA) tremendously [1]. However, some patients do not respond sufficiently to TNF inhibitors or experience side effects. Also, treatment discontinuation leads to fast relapse of disease [2, 3], and pathological osteoproliferation continues under anti-TNF treatment [4–8]. Accordingly, there is still an unmet medical need for new therapies.

Immunopathological studies on peripheral SpA (pSpA) synovitis recently identified the mast cell as potential therapeutic target. Mast cells are key players of the innate immune system and produce and secrete a variety of cytokines [9, 10]. Besides their well-established role in allergies, these cells also play an important role in rheumatoid synovitis [9–13]. We recently proposed that mast cells might be more important in SpA than in rheumatoid arthritis (RA) based on the following: [13] Firstly, the infiltration with c-Kit + mast cells in SpA synovitis is markedly higher compared to RA. Secondly, this infiltration is already observed in early disease and is not affected by effective anti-TNF treatment. Thirdly, mast cells are the major interleukin (IL)-17 expressing cells in pSpA and the proportion of mast cells expressing IL-17 is significantly higher in SpA than RA synovitis. Fourthly, proof-of-concept studies demonstrated that IL-17A blockade effectively down-modulates inflammation and clinical symptoms in the ankylosing spondylitis (AS) and psoriatic arthritis (PsA) subtypes of SpA [14–16]. Finally, sulfasalazine, the only disease-modifying anti-rheumatic drug (DMARD) with proven efficacy in pSpA [17], has shown to inhibit degranulation and TNF secretion by mast cells [18, 19].

Mast cells can be targeted in vivo by tyrosine kinase inhibitors such as imatinib and nilotinib, which are registered for the treatment of chronic myeloid leukemia (CML) [20, 21]. Originally developed to inhibit c-Abl on malignant leucocytes, these drugs also appeared to inhibit c-Kit, the receptor for stem cell factor, thereby inducing apoptosis of mast cells, including synovial mast cells [22]. Accordingly, we recently demonstrated in ex vivo biopsy tissue cultures that imatinib strongly reduced spontaneous production and secretion of pro-inflammatory cytokines including IL-6, IL-8 and IL-17 by SpA synovium [13]. In line with this data, a small open label trial with imatinib in six SpA patients showed a decrease in clinical and serum markers of disease activity upon 3 months of treatment [23].

The objective of the present study was to evaluate mast cells as potential therapeutic target in SpA by conducting a proof-of-concept randomized controlled trial with nilotinib. Nilotinib is a second-generation tyrosine kinase inhibitor which is more effective in the treatment of CML and has a better safety profile compared to imatinib [24, 25]. As the rational for tyrosine kinase inhibition is mainly based on synovial studies, we explored the immunomodulating effects on synovial histopathology, systemic inflammation, and symptoms of pSpA. Additionally, we evaluated clinical efficacy in axial SpA (axSpA) in this exploratory study.

## Methods

### Patients and study design

Twenty-eight patients diagnosed with SpA by their treating rheumatologist and fulfilling the European Spondyloarthropathy Study Group (ESSG) criteria [26] were included in a single centre, double-blind, investigator initiated clinical trial and randomized (1:1) to receive nilotinib (Tasigna; Novartis Pharmaceuticals) 400 mg twice daily or matching placebo capsules (Tiofarma B.V.) for 12 weeks, followed by an open label extension with nilotinib for another 12 weeks. The Assessment of Spondyloarthritis International Society (ASAS) criteria were not yet published when this clinical trial was designed, therefor the ESSG criteria were used in this study. Patients were considered to have pSpA when they had an arthritis, to have axSpA when they had inflammatory back pain, and combined disease if they had both an arthritis and inflammatory back pain. Patients were between 18 and 65 years old and had active disease despite treatment with non-steroidal anti-inflammatory drugs (NSAIDs). Active disease was defined as: patient’s and physician’s global assessment of disease activity of ≥40 mm, as well as ≥1 swollen and ≥1 tender joint in case of pSpA (n = 11), Bath ankylosing spondylitis disease activity index (BASDAI) of ≥4 in case of axSpA (n = 15), or both in case of combined disease (n = 2). The cohort included ten AS, ten PsA, and eight undifferentiated SpA patients. Stable doses of NSAIDs, corticosteroids (≤10 mg/day prednisone equivalent), methotrexate, sulfasalazine and leflunomide were allowed during the trial, but not intra-articular corticosteroids. Prior anti-TNF therapy (in case the reason for discontinuation was not primary failure) was permitted after a washout period (≥4 weeks in case of etanercept, ≥8 weeks in case of infliximab and ≥10 weeks for the other TNF inhibitors). All patients gave written informed consent to participate in the study as approved by the Ethics Committee of the Academic Medical Center/University of Amsterdam (Trialregister.nl registration code NTR2834). Clinical characteristics at baseline are summarized in Table 1.

**Table 1:** Baseline characteristics of the study population by treatment group

	Peripheral SpA		Axial SpA	
	Nilotinib (n = 5)	Placebo (n = 8)	Nilotinib (n = 8)	Placebo (n = 9)
Age, median (IQR) years	39.5 (37.4–52.3)	47.1 (31.8–60.4)	38.0 (36.1–42.4)	40.1 (34.5–46.7)
Disease duration, median (IQR) years	4.6 (1.0–9.4)	8.4 (4.4–11.5)	5.8 (1.8–15.3)	4.0 (1.6–12.7)
Number of men/women	3/2	4/4	7/1	6/3
Inflammatory back pain (history/presence), n (%)	0 (0.0)	3 (37.5)	8 (100.0)	9 (100.0)
Peripheral arthritis (history/presence), n (%)	5 (100.0)	8 (100.0)	3 (37.5)	3 (33.3)
Enthesitis (history/presence), n (%)	0 (0.0)	3 (37.5)	3 (37.5)	6 (66.7)
Dactylitis (history/presence), n (%)	1 (20.0)	0 (0.0)	0 (0.0)	0 (0.0)
Patient’s global assessment, median (IQR) mm	52.0 (42.5–64.5)	63.5 (50.8–82.5)	67.0 (49.8–70.8)	70.0 (59.5–86.0)
Physician’s global assessment, median (IQR) mm	46.0 (43.5–64.5)	55.5 (49.0–59.5)	56.5 (47.3–58.0)	55.0 (50.0–60.0)
66 Swollen joint count, median (IQR)	9.0 (3.0–17.5)	3.5 (1.0–8.0)	NA	NA
68 Tender joint count, median (IQR)	8.0 (0.5–19.5)	9.5 (3.5–11.8)	NA	NA
BASDAI, median (IQR)	NA	NA	5.9 (4.2–6.9)	5.4 (4.9–6.8)
CRP, median (IQR) mg/l	9.2 (0.6–29.5)	14.8 (3.0–51.7)	4.8 (3.2–5.4)	6.3 (2.6–15.2)
ESR, median (IQR) mm/h	7.0 (3.5–31.5)	10.5 (5.0–42.3)	8.5 (2.0–12.0)	16.0 (3.5–36.5)
Concomitant NSAIDs, n (%)	4 (80.0)	6 (75.0)	8 (100.0)	9 (100.0)
Concomitant corticosteroids, n (%)	0 (0.0)	0 (0.0)	0 (0.0)	0 (0.0)
Concomitant methotrexate, n (%)	2 (40.0)	4 (50.0)	2 (25.0)	0 (0.0)
Concomitant sulfasalazine, n (%)	2 (40.0)	0 (0.0)	1 (12.5)	0 (0.0)
Previous anti-TNF treatment, n (%)	1 (20.0)	2 (25.0)	2 (25.0)	2 (22.2)

Significance of the comparisons is determined by Mann–Whitney U test for continuous variables and Fisher’s exact test for categorical variables. There were no significant differences between the nilotinib and placebo groups

*SpA* spondyloarthritis; *IQR* interquartile range; *BASDAI* bath ankylosing spondylitis disease activity Index; *CRP* C-reactive protein; *ESR* erythrocyte sedimentation rate; *NSAIDs* non-steroidal anti-inflammatory drugs; *TNF* tumor necrosis factor; *NA* not applicable

### Synovial immunopathology

Synovial biopsies were obtained by mini-arthroscopy at baseline, weeks 12 and 24 in pSpA patients with active knee or ankle arthritis (n = 8) as described previously [27, 28]. Samples (6–8 per patient) were either snap-frozen in Tissue-Tek^®^ O.C.T.™ (Sakura) for histological evaluation or immediately stored in liquid nitrogen for subsequent RNA extraction and gene expression analysis.

For histopathology, cryostat sections (5 μm) were cut and mounted on Star Frost adhesive glass slides (Knittelgläser, Braunschweig). Frozen sections were acetone fixed and stained with monoclonal antibodies directed towards macrophages (CD68; EBM-11, Dako), alternatively activated macrophages (CD163; 5cFAT, BMA Biomedicals), and mast cells (c-Kit; 104D2; BioLegend). After rinsing, sections were sequentially incubated with a biotinylated secondary antibody, a streptavidin-horseradish peroxidise link, aminoethylcarbazole substrate as chromogen (all Dako), and hematoxylin as counterstain. Parallel sections were incubated with isotype and concentration-matched monoclonal antibodies as negative controls. Samples were stained in a single run to minimize technical biases. Stained sections were scored semiquantitatively for cellular infiltration by three independent observers (NY, ICB and DB) who were blinded to the patient’s treatment allocation and treatment duration, as described previously [29–31].

For gene expression analysis, mRNA was extracted using RNA Stat-60 (Tel-Test), then treated with DNase I (Invitrogen) and reverse-transcribed using a RevertAid H Minus First Strand complementary DNA Synthesis Kit (Fermentas). The RNA concentration was determined with a NanoDrop spectrophotometer. Analysis of mRNA by qPCR was performed using a StepOnePlus Real-Time PCR System (Applied Biosystems) using GAPDH as housekeeping gene. Predesigned TaqMan probe and primer sets for IL-6 (Hs00174131_m1), IL-8 (Hs00174103_m1), TNF (Hs00174128_m1), IL-17A (Hs00174383_m1), IL-17F (Hs00369400_m1), IL-23 (Hs00372324_m1), c-Kit (Hs00174029_m1), and GAPDH (4310884E) were assayed according to the manufacturer’s protocol (Applied Biosystems).

### Serum biomarkers

Serum analysis included safety measurements (liver function, renal function, blood cell counts) as well as C-reactive protein (CRP) and erythrocyte sedimentation rate (ESR). Serum levels of matrix metalloproteinase-3 (MMP-3, Biotrak, Amersham Pharmacia Biotech) and calprotectin (Hycult Biotech), two potential biomarkers of inflammation in SpA [32, 33], were measured by enzyme-linked immunosorbent assays (ELISAs) according to the manufacturer’s instructions.

### Clinical assessments

Clinical assessments consisted of safety evaluation (consisting of the patient’s history, physical examination, laboratory tests, urinalysis and electrocardiograms), patient’s and physician’s global assessment of disease activity on a visual analogue scale, ankylosing spondylitis disease activity score (ASDAS) and ASDAS improvement criteria [34, 35] in all patients. This was complemented by swollen and tender joint count (SJC66 and TJC68) in case of pSpA, and BASDAI and BASDAI50 response in case of axSpA [36, 37].

### Statistical analysis

Data are presented as the median and interquartile range (IQR). pSpA and axSpA were analyzed separately as the rational for tyrosine kinase inhibition is mainly based on synovial studies in pSpA and the assessment in axSpA was more exploratory. In case of combined SpA the patient’s data were included in both groups. The nilotinib and placebo group were compared to each other by Mann–Whitney U tests for continuous variables and Fisher’s exact test for categorical variables. Differences between various time points were assessed by Wilcoxon matched pairs tests by treatment group. For biological data (synovial immunopathology and systemic biomarkers) the weeks 0–12 data of the original nilotinib group were pooled with the weeks 12–24 data of the original placebo group (which was treated with nilotinib from week 12 onwards) to increase the power of these proof-of-concept analyses. The clinical data, which are more sensitive to placebo effects, were not pooled but analyzed as observed with the last observation carried forward. *P* values of <0.05 were considered statistically significant, and *p* values <0.1 and ≥0.05 were considered to represent a trend.

## Results

### Immunomodulation of synovial inflammation by nilotinib treatment

**Fig. 1:**
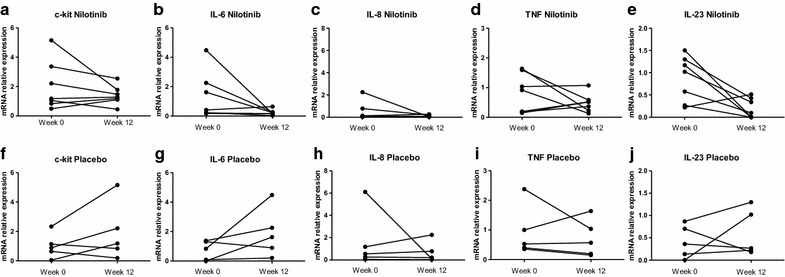
Synovial tissue mRNA expression in peripheral spondyloarthritis. Effect of nilotinib and placebo treatment on in vivo synovial tissue mRNA expression in peripheral spondyloarthritis as assessed by quantitative polymerase chain reaction. The panel represents the transcription of c-Kit, interleukin-6 (IL-6), IL-8, tumor necrosis factor (TNF), and IL-23 before and after treatment with nilotinib (**a**–**e**) or placebo (**f**–**j**). The *lines* connect the data points for each patient between weeks (week) 0 and 12

To assess the modulation of synovial inflammation by nilotinib, synovial biopsies were obtained before and after treatment in pSpA patients with knee or ankle arthritis. Since the number of snap-frozen biopsies which passed quality control for immunohistological analysis was small (n = 3 in the nilotinib group and n = 4 in the placebo group) we only used descriptive analyses for this part of the analysis. As shown in Table 2, the number of infiltrating CD68+ and CD163+ macrophages, which are markers for synovial inflammation in SpA [29, 30, 38], numerically decreased in both the lining layer and the synovial sublining upon nilotinib treatment. In contrast, there was no consistent modulation of synovial macrophage numbers between baseline and week 12 of placebo treatment. Nilotinib treatment also decreased the number of c-Kit + synovial mast cells, while there was a numerical increase after placebo treatment. This was confirmed by qPCR analysis of mRNA expression, as c-Kit expression showed a significant decrease upon nilotinib treatment but augmented upon placebo treatment over 12 weeks (p = 0.037) (Fig. 1a). Additionally, nilotinib treatment induced a decrease of the synovial mRNA expression of the pro-inflammatory cytokines IL-6 (p = 0.024) and IL-23 (p = 0.024) compared to placebo, but not of IL-8 (p = 0.378) and TNF (p = 0.500) (Fig. 1b–e). Expression levels of IL-17A and IL-17F were too low (even in the baseline biopsies) to allow reliable detection.

**Table 2:** Immunomodulatory effect of nilotinib versus placebo treatment on synovial histopathology

Marker	Nilotinib		Placebo	
	Week 0	Week 12	Week 0	Week 12
CD68 lining	1.7 (1.7–3.0)	0.3 (0.0–2.0)	2.8 (1.2–3.0)	2.3 (1.7–3.0)
CD68 sublining	2.0 (1.3–2.3)	0.0 (0.0–1.3)	2.0 (1.0–3.0)	1.7 (1.3–2.0)
CD163 lining	2.7 (2.0–2.7)	0.7 (0.0–2.0)	2.8 (0.7–3.0)	2.7 (2.7–2.7)
CD163 sublining	2.3 (2.3–2.7)	1.3 (0.3–2.0)	2.3 (1.4–3.0)	2.3 (2.3–2.3)
c-Kit	1.0 (0.3–2.3)	0.0 (0.0–0.7)	0.0 (0.0–0.8)	1.3 (0.3–2.3)

Values are the median (IQR) assessed on a semiquantitative scale

### Modulation of systemic inflammation by nilotinib treatment in pSpA

**Fig. 2:**
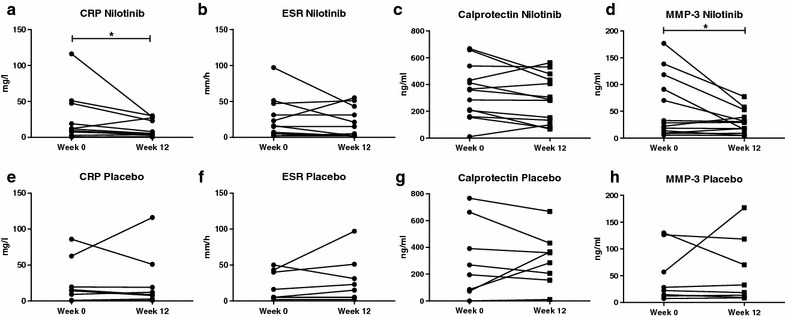
Serum biomarkers in peripheral spondyloarthritis. Effect of nilotinib and placebo on serum biomarkers of patients with peripheral spondyloarthritis. The *panel* represents the C-reactive protein (CRP), erythrocyte sedimentation rate (ESR), calprotectin and matrix metalloproteinase-3 (MMP-3) levels before (week 0) and after treatment (week 12) with nilotinib (**a**–**d**) or placebo (**e**–**h**). The *lines* connect the data points for each patient. *P value <0.05

As the tissue analysis indicated that nilotinib down-regulates synovial inflammation, we next examined whether systemic biomarkers of inflammation are also modulated by nilotinib in pSpA (Fig. 2). CRP remained stable in the placebo group but decreased significantly from 9.2 (IQR 1.7–33.1) to 5.2 (IQR 1.7–25.1) mg/l (p = 0.024) upon 12 weeks of nilotinib treatment. This was particularly marked in patients with high levels at baseline. CRP levels decreased even further after 24 weeks of nilotinib treatment to 4.0 (IQR 0.4–25.5) mg/l (p = 0.031 compared to baseline). The effect on ESR (p = 0.141) was less clear. Calprotectin showed a trend towards improvement after 12 weeks treatment with nilotinib decreasing from 359.9 (IQR 183.3–484.9) to 287.9 (IQR 116.7–457.1) ng/ml (p = 0.055), but stayed stable in the placebo group. MMP-3 also mainly decreased in patients with high levels at baseline, but overall the median showed a slight increase after 12 weeks of nilotinib from 28.5 (IQR 11.6–104.8) to 29.3 (IQR 12.3–46.0) ng/ml (p = 0.034).

### Improvement of clinical symptoms after nilotinib treatment in pSpA

**Fig. 3:**
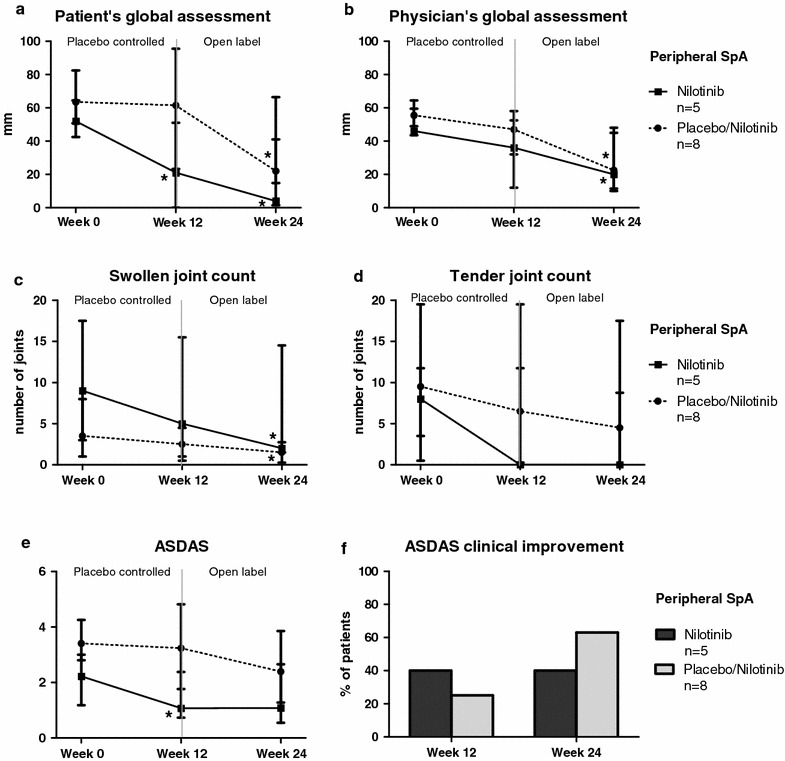
Clinical disease activity in peripheral spondyloarthritis. Changes in clinical disease activity parameters during treatment with nilotinib or placebo from week 0 until week 12, and during the open label extension phase with nilotinib from week 12 until week 24 in patients with peripheral spondyloarthritis. The *panel* represents the median (interquartile range) in patient’s global assessment of disease activity (**a**), physician’s global assessment of disease activity (**b**), swollen joint count (**c**), tender joint count (**d**), ankylosing spondylitis disease activity score (ASDAS) (**e**), and the percentage of patients achieving ASDAS clinically important improvement (**f**). *P value <0.05 compared to baseline

In line with the synovial tissue and serum biomarker analyses nilotinib treatment induced a significant reduction in patient’s global assessment at week 12 (p = 0.031) as well as after an additional 12 weeks of open label treatment with nilotinib (p = 0.031) (Fig. 3a). Moreover, the placebo treated patients did not show any changes in patient’s global assessment during the first phase of the study but showed significant improvement after entering the open label phase with nilotinib treatment (p = 0.012) (Fig. 3a). The physician’s global assessment showed a trend towards improvement at week 12 (p = 0.063) and improved significantly at week 24 (p = 0.031) (Fig. 3b). The SJC66 and TJC68 decreased numerically between week 0 and 12 of nilotinib but not placebo treatment and decreased further at week 24, which was significant for the SJC66 (p = 0.049). After entering the open label phase with nilotinib the originally placebo treated patients also showed significant improvement in SJC66 (p = 0.031) (Fig. 3c, d). The ASDAS, a composite measure originally developed for axSpA but which was also shown to be useful in pSpA [39], improved after 12 weeks of nilotinib treatment (p = 0.031), which was not the case for placebo (p = 0.371) (Fig. 3e). ASDAS clinically important improvement was reached by 40.0 % of the nilotinib group at week 12 (Fig. 3f) and by 53.8 % of the total study population at week 24.

### Lack of effect of nilotinib treatment in axSpA

**Fig. 4:**
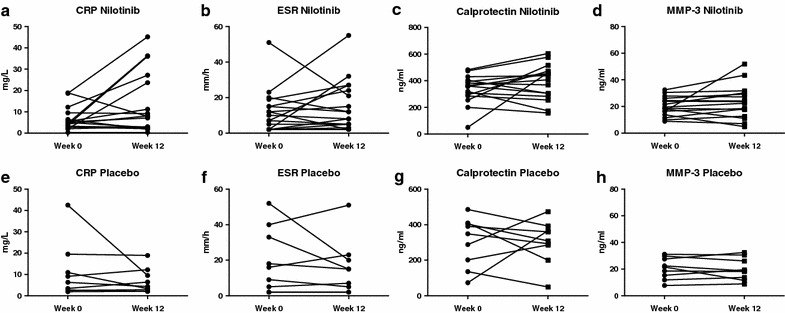
Serum biomarkers in axial spondyloarthritis. Effect of nilotinib and placebo on serum biomarkers of patients with axial spondyloarthritis. The *panel* represents the C-reactive protein (CRP), erythrocyte sedimentation rate (ESR), calprotectin and matrix metalloproteinase-3 (MMP-3) levels before (week 0) and after treatment (week 12) with nilotinib (**a**–**d**) or placebo (**e**–**h**). The *lines* connect the data points for each patient. *P value <0.05

**Fig. 5:**
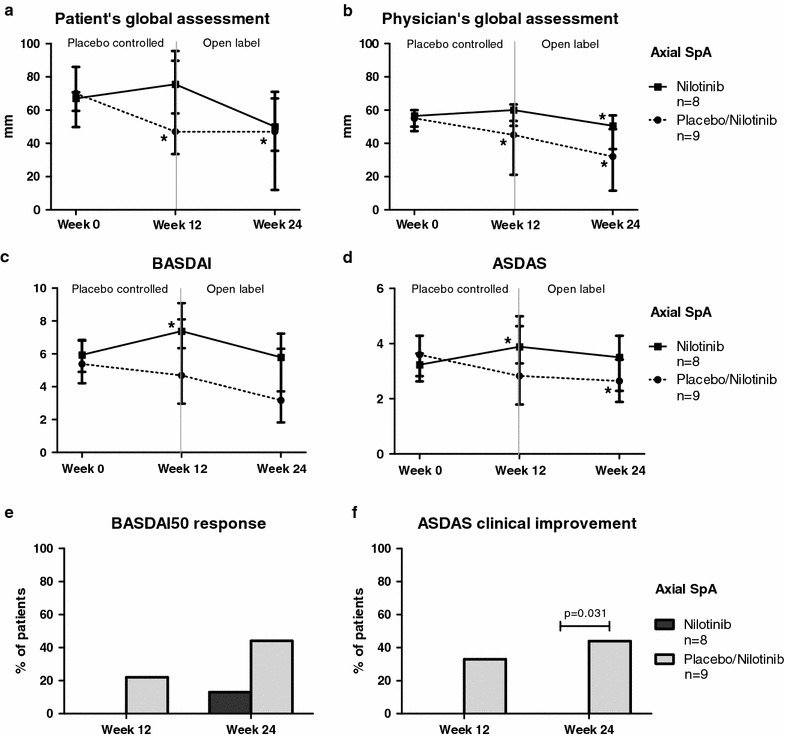
Clinical disease activity in axial spondyloarthritis. Changes in clinical disease activity parameters during treatment with nilotinib or placebo from week 0 until week 12, and during the open label extension phase with nilotinib from week 12 until week 24 in patients with axial spondyloarthritis. The *panel* represents the median (interquartile range) in patient’s global assessment of disease activity (**a**), physician’s global assessment of disease activity (**b**), bath ankylosing spondylitis disease activity index (BASDAI) (**c**), ankylosing spondylitis disease activity score (ASDAS) (**d**), and the percentage of patients achieving a BASDAI50 response (**e**), and ASDAS clinically important improvement (**f**). *P value <0.05 compared to baseline

Although the rational to study nilotinib treatment in SpA was based on synovial tissue findings in pSpA, we also explored the effect of nilotinib in axSpA since the published open label trial with imatinib also showed improvement in axial symptoms [23]. In contrast with pSpA, however, serum CRP (baseline 4.2 (IQR 2.9–8.0) mg/l versus week 12 7.8 (IQR 2.4–25.5) mg/l, p = 0.054) as well as the other tested systemic biomarkers of inflammation did not show a reduction but even an increase after nilotinib treatment (Fig. 4). More importantly, the clinical parameters did not improve upon nilotinib treatment, while the placebo response was unexpectedly high for this patient group (Fig. 5). Patient’s and physician’s global assessment were unchanged at week 12 for the nilotinib arm but, surprisingly, was significantly decreased in the placebo group (from 70 (IQR 60–86) to 47 (IQR 34–96) mm, p = 0.046, and from 55 (IQR 50–60) to 45 (IQR 21–54) mm, p = 0.010, respectively). BASDAI (from 5.9 (IQR 4.2–6.9) to 7.4 (IQR 6.3–8.1), p = 0.039) and ASDAS (from 3.2 (IQR 2.6–3.6) to 3.9 (IQR 3.3–5.0), p = 0.020) were even increased at week 12 in the nilotinib treated patients. ASDAS clinically important improvement at week 12 was reached more often in the placebo group (33.3 %) than in the nilotinib group (0 %) (p = 0.072), with a similar pattern for BASDAI50 response (22.2 versus 0 % respectively, p = 0.156). This was even more striking at week 24 in which ASDAS clinically important improvement was reached in 44.4 % of the placebo treated patients compared to 0 % in the nilotinib group (p = 0.031), with again a similar pattern for the BASDAI50 response (44.4 versus 12.5 % respectively, p = 0.149). Collectively, these data indicate a lack of effect of nilotinib on axSpA.

### Safety analysis

Although the present study is too small in size and duration to come to stringent safety conclusions, there were no unexpected safety signals in comparison with already available large scale data in CML [24, 25]. The overall number of adverse events (AEs) was high in both the nilotinib and placebo group (Table 3). The most common AEs were headache, dermatologic conditions (rash or acne), and gastrointestinal complaints (stomach ache or nausea). In contrast to the CML trials, haematological and biochemical AEs occurred rarely (anemia, thrombocytopenia, hypomagnesemia, and lipase elevation occurred in one patient each). Significant prolongation of the QT-interval on the electrocardiogram did not occur. All AEs were transient but dose reduction (from 400 mg twice daily to 400 mg once daily) was needed in seven patients (mostly temporarily), after which the AEs resolved. One patient developed an acute cholecystitis at week 21 for which he was hospitalized; this was considered a serious adverse event unrelated to the study drug.

**Table 3:** AEs possibly related to nilotinib, occurring in more than one patient and all SAEs

	Nilotinib (n = 13)	Placebo/nilotinib (n = 15)	
	Week 0–24	Week 0–12	Week 12–24
Number of patients with AEs	13 (100.0)	14 (93.3)	13 (86.7)
Infections			
Common cold	5 (38.5)	4 (26.7)	4 (26.7)
Gastroenteritis	4 (30.8)	2 (13.3)	1 (6.7)
Sinusitis	2 (15.4)	0 (0.0)	0 (0.0)
Dermatological			
Rash/acne	8 (61.5)	2 (13.3)	6 (40.0)
Hair loss	3 (23.1)	2 (13.3)	2 (13.3)
Mastopathy	2 (15.4)	0 (0.0)	1 (6.7)
Gastrointestinal			
Stomach ache	5 (38.5)	3 (20.0)	1 (6.7)
Nausea	5 (38.5)	3 (20.0)	6 (40.0)
Anorexia	2 (15.4)	3 (20.0)	2 (13.3)
Weight loss	2 (15.4)	1 (6.7)	1 (6.7)
Constipation	3 (23.1)	1 (6.7)	0 (0.0)
Cardiological			
Palpitations	1 (7.7)	0 (0.0)	2 (13.3)
Musculoskeletal			
Myalgia	3 (23.1)	0 (0.0)	2 (13.3)
Peripheral edema	2 (15.4)	1 (6.7)	0 (0.0)
Flank pain	3 (23.1)	0 (0.0)	0 (0.0)
Neurological			
Headache	5 (38.5)	1 (6.7)	2 (13.3)
Ear/nose/throat			
Cough	0 (0.0)	0 (0.0)	2 (13.3)
Psychological			
Depressive feelings	2 (15.4)	0 (0.0)	0 (0.0)
Other			
General malaise	1 (7.7)	0 (0.0)	1 (6.7)
Tiredness	3 (23.1)	1 (6.7)	2 (13.3)
Hot flushes	1 (7.7)	0 (0.0)	2 (13.3)
Sicca	0 (0.0)	1 (6.7)	2 (13.3)
Number of patients with SAEs	1 (7.7)	0 (0.0)	0 (0.0)
Hospital admission	1 (7.7)	0 (0.0)	0 (0.0)

Values are the number (percentage) of patients. The SAE concerned one case of hospital admission following laparoscopic surgery because of acute cholecystitis. The placebo/nilotinib treated patients received placebo from week 0 until week 12, and nilotinib from week 12 until week 24

*AE* adverse event; *SAE* serious adverse event

## Discussion

We recently proposed that mast cells may contribute to SpA synovitis by indicating that the number of infiltrating mast cells was specifically increased in SpA versus RA synovitis, SpA synovial mast cells contained IL-17A as assessed by immunostaining, and ex vivo targeting of mast cells with imatinib reduced inflammation in synovial biopsy cultures [13]. The present study supports the role of mast cells in synovial inflammation by demonstrating histological, biological and clinical effects of nilotinib treatment in pSpA. Tyrosine kinase inhibitors such as nilotinib and imatinib target c-Kit, which is crucial for the survival of mast cells. In agreement with previous ex vivo studies demonstrating that imatinib induces apoptosis of synovial mast cells [22], in vivo treatment with nilotinib induced a decrease in the number of synovial mast cells and in c-Kit mRNA expression in pSpA. This was associated with a decrease in infiltrating macrophages, synovial expression of pro-inflammatory cytokines such as IL-6, systemic CRP levels, and clinical disease activity parameters. Importantly, the immunomodulatory effect of nilotinib was consistent across biological and clinical measurements and was not observed in the placebo group. Together with the published small open label study with imatinib [23], these data indicate that tyrosine kinase inhibitors targeting mast cells appear to be able to suppress inflammation in pSpA and thereby support the potential role of mast cells in SpA pathogenesis.

Due to the proof-of-concept design, this study has limitations which should be considered when interpreting the data. Firstly, the number of pSpA patients in the double-blind phase was small, particularly in the synovial evaluation as the quality of the synovial biopsies was insufficient in a number of patients. Therefore the data for the biological parameters were pooled (weeks 0–12 of the originally nilotinib treated patients with weeks 12–24 of the patients receiving placebo originally and subsequently nilotinib). Secondly, the week 12 primary endpoint was perhaps too short. Indeed, both CRP and clinical parameters tended to decrease even further after an additional 12 weeks of treatment. Thirdly, the study did not allow to define mechanistically how nilotinib exerted its immunomodulatory effects. Even though our analysis suggested an effect on synovial IL-23 but not TNF expression, the number of good quality synovial biopsies was too small to determine reliably which inflammatory pathways are or are not modulated by nilotinib. For example, mRNA expression of key cytokines such as IL-17A and IL-17F was too low for reliable analysis. Furthermore, c-Kit is not only expressed on mast cells but on other immune cells as well, including innate lymphoid cells, which can express and produce pro-inflammatory mediators [40]. Moreover, nilotinib can also target other tyrosine kinases such as c-Fms and platelet derived growth factor receptor (PDGF-R). C-Fms is expressed on CD163+ macrophages, which are significantly increased in SpA synovitis [29, 41, 42], and PDGF-R is a key molecule on myofibroblasts, which were recently shown to be specifically increased in SpA versus RA synovitis [43]. Since targeting mast cells, innate lymphoid cells, CD163+ macrophages, as well as myofibroblasts all might be beneficial in pSpA, it remains unknown how nilotinib actually reduces synovial and systemic inflammation. Finally, the potency of nilotinib compared to other treatments for pSpA such as sulfasalazine [17] or TNF blockade [39] is unknown, as we did not include an active comparator group.

Strikingly, the biological and clinical effects observed in pSpA were completely absent in axSpA. This is in agreement with the concept that peripheral and axSpA might be driven by slightly distinct cellular and molecular mechanisms [44]. For example, the major cellular source of IL-17 in pSpA are mast cells and to a lesser degree neutrophils [13], while in axSpA neutrophils and myeloperoxidase (MPO)+ cells are the major IL-17 expressing cells [45]. The discrepant response to nilotinib between pSpA and axSpA is also in line with previous data with sulfasalazine, which also targets mast cells [18, 19] and has proven clinical efficacy in peripheral but not axial disease [17]. Taken together, the histopathology and the studies with sulfasalazine and nilotinib point towards partially distinct inflammatory pathways in peripheral versus axial disease. However when interpreting these data, it must be considered that it was not the intention of the current trial to compare the efficacy of nilotinib between pSpA and axSpA, but between nilotinib and placebo in an exploratory fashion, hence pSpA and axSpA were not compared to each other. The effect of nilotinib on enthesitis, dactylitis and extra-articular manifestations (psoriasis, inflammatory bowel disease, uveitis) of SpA remains to be investigated.

A final observation was the pronounced placebo response in axSpA, which was unusually high compared to what is commonly reported [46–48]. Although we do not have a clear explanation for this finding, this observation indicates that open label trials in axSpA should be interpreted with caution and pleas for a double-blind placebo-controlled arm not only in large phase III trials but also in proof-of-concept trials.

## Conclusions

This proof-of-concept study supports the concept that mast cells can contribute to synovial inflammation in SpA and that tyrosine kinase inhibition targeting these cells has a biological and clinical immunomodulatory effect in pSpA. A similar response was not observed in axSpA in this small exploratory trial. These results support further clinical evaluation of nilotinib in larger clinical trials in pSpA as well as evaluation of other drugs targeting mast cells in SpA.

## Abbreviations

AE: adverse event
AS: ankylosing spondylitis
ASAS: Assessment of Spondyloarthritis International Society
ASDAS: ankylosing spondylitis disease activity score
axSpA: axial spondyloarthritis
BASDAI: bath ankylosing spondylitis disease activity index
CML: chronic myeloid leukemia
CRP: C-reactive protein
IL: interleukin
IQR: interquartile range
DMARD: disease modifying anti-rheumatic drug
ELISA: enzyme-linked immunosorbent assays
ESR: erythrocyte sedimentation rate
ESSG: European Spondyloarthropathy Study Group
MMP: matrix metalloproteinase
MPO: myeloperoxidase
NSAID: non-steroidal anti-inflammatory drug
PDGF-R: platelet derived growth factor receptor
PsA: psoriatic arthritis
pSpA: peripheral spondyloarthritis
RA: rheumatoid arthritis
SJC: swollen joint count
SpA: spondyloarthritis
TJC: tender joint count
TNF: tumor necrosis factor
